# Low-Energy Data Collection in Wireless Sensor Networks Based on Matrix Completion

**DOI:** 10.3390/s19040945

**Published:** 2019-02-23

**Authors:** Yi Xu, Guiling Sun, Tianyu Geng, Jingfei He

**Affiliations:** 1 College of Electronic Information and Optical Engineering, Nankai University, Tianjin 300071, China; xuyi@mail.nankai.edu.cn (Y.X.); 1010337@mail.nankai.edu.cn (T.G.); 2 Department of Key Laboratory of Electronic Materials and Devices of Tianjin, School of Electronics and Information Engineering, Hebei University of Technology, Tianjin 300401, China; hejingfei@hebut.edu.cn

**Keywords:** wireless sensor networks, data collection, sparse sampling, matrix completion

## Abstract

Sparse sensing schemes based on matrix completion for data collection have been proposed to reduce the power consumption of data-sensing and transmission in wireless sensor networks (WSNs). While extensive efforts have been made to improve the recovery accuracy from the sparse samples, it is usually at the cost of running time. Moreover, most data-collection methods are difficult to implement with low sampling ratio because of the communication limit. In this paper, we design a novel data-collection method including a Rotating Random Sparse Sampling method and a Fast Singular Value Thresholding algorithm. With the proposed method, nodes are in the sleep mode most of the time, and the sampling ratio varies over time slots during the sampling process. From the samples, a corresponding algorithm with Nesterov technique is given to recover the original data accurately and fast. With two real-world data sets in WSNs, simulations verify that our scheme outperforms other schemes in terms of energy consumption, reconstruction accuracy, and rate. Moreover, the proposed sampling method enhances the recovery algorithm and prolongs the lifetime of WSNs.

## 1. Introduction

Wireless Sensor Networks (WSNs) have attracted many researchers’ attention and have been deeply studied [1]. With the growing demand for big data, the size of WSNs is increasing, which raises greater challenges to data-collection technologies of WSNs. Moreover, the control of power consumption at the sensor nodes is always a main problem faced by WSNs. To solve the problem, sparse sensing schemes as efficient methods for data collection have been proposed, optimizing the overall energy consumption. It reduces the power consumption of data sensing and data transmission, prolonging the network life.

Sparse sensing schemes concentrate on the reduction of the number of samples and the reconstruction of the missing data using algorithms exploiting the data structure available in the meantime. Based on the sparsity of sensor observations, Compressed Sensing (CS) theory [2] has been used to compress and reconstruct the data. Although CS theory has been widely used in WSNs [3,4,5], the number of samples is not actually reduced because sensor nodes need to sample all data and then compress. Later according to the research progress of the Matrix Completion (MC) theory [6], researchers exploited the low-rank characteristic of sensor data and applied the MC theory to WSNs [7]. In MC, the low-rank data matrix can be recovered accurately with some samples uniformly at random [6], which means only a portion of data are needed to be sensed and transmitted to the sink, realizing a real sense of sparse sensing.

The data-collection methods based on MC theory mainly include the sampling method and the recovery algorithm. It can reduce the energy consumption from two aspects. One is from reducing the amount of data being sensed and transmitted, and the other one is that many sensor nodes are in the sleep mode when they are not selected to sense data. However, this has a strict requirement for the topology of WSNs. When some nodes are in the sleep mode, the sink may not be able to receive the sensed data due to the communication limits, especially in large-scale WSNs and many realistic environments. Even though the topology can be set up adaptively, it will be energy-consuming and time-consuming as the active nodes are varying. The above problems are ignored by many sampling methods and corresponding algorithms based on MC theory.

Since the application of MC theory in WSNs, many algorithms for solving the MC problem are adapted to WSNs. In the beginning, people use the alternative least squares (ALS) method to solve the data recovery problem exploiting the spatial correlation of WSNs data, which is called the Efficient Data-Collection Approach (EDCA) [7]. Later the Spatio-Temporal Compressive Data Collection (STCDG) [8] was proposed adding the short-term stability features to improve the accuracy. These methods assume the rank of the data is fixed and known, which is unlikely to hold in the practical system. He et al. combined the MC with sparsity constraints, proposing the data recovery method with joint MC and Sparsity Constraints (DRMCSC) [9], and used the alternating minimization to solve the problem.

The recovery methods of previous works make the reconstruction accuracy of WSNs data high; however, the models and the alternative optimization algorithms used in previous works are usually complex and cost a large amount of running time. With the number of data increasing, the recovery efficiency will be a problem. Singular Value Thresholding (SVT) algorithm is a classic algorithm for solving the MC problem quickly [10]. However, the recovery accuracy of SVT algorithm cannot be guaranteed for all kinds of original data and samples. The samples of most previous data-collection methods are obtained randomly with a deterministic sampling ratio. Practical and efficient sampling methods have been proposed such as the adaptive sampling with MC for specific real WSNs data [11], determining the effective sampling set with three sample learning principles and dealing with the corrupted problem [12]. However, the sampling method is complex and energy-consuming.

We assume the WSN is large-scale and monitors slowly changing environment parameters over time such as the temperature, the humidity, and the light. Moreover, the sensor nodes sense the data at set intervals and the interval is short, usually several to dozens of seconds. This scenario usually exists in agriculture and environmental protection field. With these assumptions, to recover the data accurately and fast and to reduce the energy consumption in WSNs, we propose a low-energy data-collection method based on sparse sensing with MC theory in this paper. Our method includes a Rotating Random Sparse Sampling (RRSS) method and a Fast Singular Value Thresholding (FSVT) algorithm. The proposed method can be used not only for WSNs but also in the context of more complex networks as Wireless Sensor and Actuator Networks (WSANs) [13,14] or the Wireless Sensor, Actuator and Robot Network (WSARN) [15]. The main contributions of this paper are summarized as follows:

- We propose a low-energy data-collection method for large-scale WSNs including a load-balancing random sampling method and a corresponding algorithm to recover the original data from the samples accurately and fast. The data-collection method can be combined with any topologies of WSNs easily without the limit to communication change in sparse sensing schemes.
- We design a rotating varying sampling ratio based on sparse sampling and set a time schedule, on which sensor nodes are in the sleep mode most of the time to greatly reduce the energy consumption.
- We construct the model with Tikhonov regularization for recovering the original signal from the samples and use the SVT algorithm with the Nesterov technique to solve it. Theoretical explanations of the effectiveness of the algorithm have been given.
- With two real-world data sets, we evaluate the proposed sampling method with the corresponding FSVT algorithm compared the traditional sampling method and other data-collection methods. The simulation results show that the proposed sampling method can enhance the FSVT algorithm and the recovery performance outperforms other methods in terms of the reconstruction accuracy and rate.

The rest of this paper is organized as follows. Section 2 presents the basic collection method with matrix completion for WSNs including the basic MC theory and the random sampling method. The proposed sampling method is described in Section 3. According to the proposed sampling method, the problem formulation and the solution are given in Section 4. Section 5 presents the experiments results of the proposed methods compared with the state-of-the-art methods in the term of reconstruction accuracy and running time. Finally, Section 6 concludes the paper.

## 2. Data Collection Based on MC

In a WSN system, considering *N* sensor nodes and one sink, sensor nodes sense and transmit the signal to the sink. We define a matrix $X_{N\times T}$ to represent the original data from *N* sensor nodes over *T* time slots without loss of generality. In the data matrix *X*, the row index corresponds to the node index and the column index corresponds to the time slot so that an element $X_{ij}$ represents the data for a specific sensor node *i* in the $j^{th}$ time slot.

MC is to solve the problem of recovering a data matrix from a few samplings of its entries. Candes et al. [6] proved that if the number *m* of sampled entries obeys

(1) $$m>>Cn^{1.2}r\log n$$

for some positive numerical constant *C*, then with very high probability, most n×n matrices of rank *r* can be perfectly recovered by solving a simple convex optimization problem.

Many data-collection methods for WSNs are developed based on the MC theory [7,8,9]. For these methods, at each time slot, some sensor nodes are selected to sense data randomly with a deterministic sampling ratio and then transmit the data to the sink. After *T* time slots, the sink gets the measurements matrix, which is generally incomplete. Only the positions corresponding to the selected nodes offer valid sensing data. The values in the other positions are usually set as zeros. Here we adopt a linear operator A(·) to represent the sampling procedure:

(2) A(X)=M

where *X* is the original data without the loss of generality and *M* is the incomplete measurements matrix. It can be also seen as a Hadamard product of *X* and a sampling matrix, which is linear [16]. As this operator is linear, it is easy to recover *X* from *M* with the constraint of Equation (2). Previous studies have shown that data collected from WSNs are highly spatial and temporary correlated [17], which brings the low-rank characteristic of the matrix *X*. According to the work of the MC theory [6], it is highly possible to recover a low-rank matrix from a subset of its entries. Thus, the data recovery problem can be formulated as follows:

(3) $$\begin{array}{cc} \underset{X}{\mathrm{minimize}} & \mathrm{rank}(X)\\ \mathrm{subject}\;\mathrm{to} & A(X)=M. \end{array}$$

**Figure 1:**
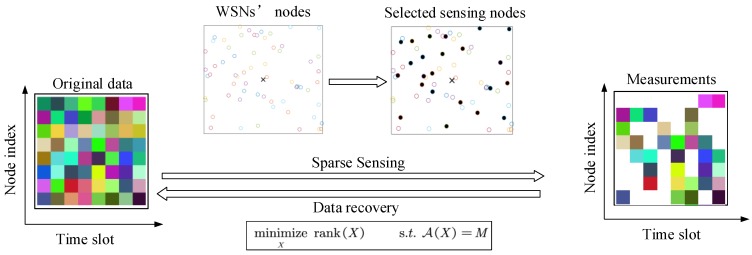
The data-collection method based on matrix completion.

The brief graphical representation for the data-collection method based on MC is shown in Figure 1. Circles represent the nodes and the × in the middle represents the sink. Through the selected sensing nodes, which are shown as black circles, the sink gets the measurements matrix and then recovers the original data according to the Equation (3). Because only a portion of sensor nodes need to sense and transmit data, the energy consumption has been reduced and the lifetime of WSNs has been prolonged.

## 3. The Rotating Random Sparse Sampling Method

From the description of the data-collection method based on MC theory, it can be known that the original data can be recovered from the samples. However, the positions of the selected sensing nodes have great impacts on the recovery performance and the energy consumption of WSNs. Although the previous random sampling method ensures the above two aspects to an extent, it has some difficulties in implementing it considering the communication limit when random nodes are in the sleep mode. Moreover, it may generate all-zeros column in the measurements matrix when the sampling ratio is low, which poses a challenge to the recovery algorithm.

We design the RRSS method based on MC. The random characteristic of the sampling method remains to ensure the security of WSNs. In contrast to the previous random sampling method, the sampling ratio is varying over time slots and it may be different among sensor nodes.

We define a round include a transmission period and *c* time slots, during which sensor nodes sense. Each round every sensor node only samples once and transmits once so that the overall sampling ratio is p=1/c for the whole WSN. With these definitions, the proposed RRSS method is explained as follows in chronological order.

At the beginning of each round, which is also called the transmission period, all sensor nodes will wake up independently, set up the topology and transmit the data collected last round. *N* data will be collected at the sink as each sensor node sense once in a round. The data package of each sensor node includes the data value *x*, the number of sensing time slot *j* and the node ID *i* so that data value *x* can be formed as $X_{ij}$ at the sink. It is noteworthy that our method can be easily combined with any WSN topology and data compression method because all sensor nodes are active when transmitting and the number of data each sensor node transmits is deterministic.

After the transmission period, during the first time slot of each round, each sensor node *i* chooses a uniform random number $a_{iu}$ between 0 and 1 at the $u^{th}$ round and then compares $a_{iu}$ with a threshold. The sensor node is selected to sense if its random number $a_{iu}$ is less than the threshold. The threshold is related to the sparse sampling ratio *p* and defined as follows:

(4) $$P_{i}\left(j\right)=\frac{p}{1-p\times ((j-1)\;\mathrm{mod}\;c)}\;,\;i\in G_{u},$$

where j=1,2,...T. u=ceil(j/c) is the nearest integer greater than or equal to (j/c) and represents the number of current round. We define the set $G_{u}$ in which sensor nodes have not been selected to sense in the $u^{th}$ round. The explanation of this definition is given later in this section after the chronological description.

The selected nodes sense and send their sensed data to the sink during the $j^{th}$ time slot, then it will not belong to the set $G_{u}$ anymore. After that, these selected nodes will fall into sleep mode during the remaining time slots of this round to reduce energy consumption.

In the following time slots of this round, the nodes $i\in G_{u}$ repeat the process of random number generation and comparison with the threshold as well as falling into sleeping mode after being selected to sense.

**Figure 2:**
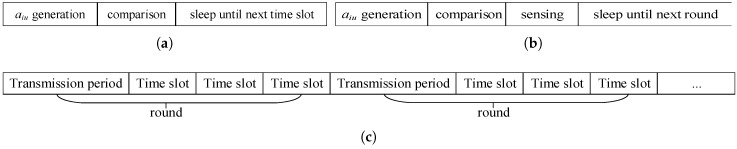
Time scheduling. (**a**) A time slot of non-sensing nodes. (**b**) A time slot of sensing nodes. (**c**) Time scheduling of the network when c=3.

When re-entering a new round, all nodes will wake up and start the data transmission and the random number generation process, repeating the same sampling process as the previous round. The time scheduling of the process is shown in Figure 2.

Because a round includes *c* time slots, the mean number of selected nodes during each time slot is N/c=N×p, which we define as *r* so that Equation (4) can be transformed into

(5) $$P_{i}\left(j\right)=\frac{r}{N-r\times ((j-1)\;\mathrm{mod}\;c)},\;i\in G_{u}.$$

It can be seen that the probability $P_{i}\left(j\right)$ changes with the time slot varying, which makes the sampling ratio keep *p* and the number of selected nodes keep *r*. Because each node only senses once in a round, the number of nodes in the set $G_{u}$ reduces and can be formulated as N(j)=N−r×((j−1)modc) so that the expected number of selected sensor nodes during each time slot is:

(6) $$\begin{array}{cc} E & =N\left(j\right)\times P_{j}\\ & =\left(N-r\times \left(\left(j-1\right)\;\mathrm{mod}\;c\right)\right)\times \frac{r}{N-r\times ((j-1)\;\mathrm{mod}\;c)}\\ & =r. \end{array}$$

Because the method for selecting sensor nodes to sense and to transmit is rotating and random, and nodes will fall into the sleep mode after sensing once in a round, the RRSS method can reduce and balance the energy consumption of the network as well as sampling randomly and sufficiently to make the system secure and the recovery performance more accurate and fast.

It is notable that the proposed method is suitable for monitoring slowly changing environment parameters over time. The time length of a round is usually several minutes. The time length of a transmission period $t_{r}$ is according to the size of WSNs as it needs to transmit all data to the sink. The time length of a time slot $t_{s}$ can be several to dozens of seconds as described above. So, the time length of a round can be expressed as $\left(t_{r}+t_{s}\times c\right)$.

## 4. Problem Formulation and the Solution

Using the RRSS method, the sink obtains the observed data defined as $B_{N\times T}$. We use a sampling matrix $Q_{N\times T}$ of which the element is 1 when the data is observed or 0 otherwise. According to Equation (4), *Q* is defined as follows in detail:

(7) $$Q=\left(\begin{array}{lll} \;0\;1\;0\;\cdots & 0\;\cdots \;0\;1 & \cdots \\ \;0\;\cdots \;1\;0 & \cdots \;1\;0\;0 & \cdots \\ \;\vdots \;\vdots \;\vdots \;\vdots & \;\vdots \;\vdots \;\vdots \;\vdots & \;\vdots \\ \;0\;1\;0\;\cdots & \;0\;\cdots \;1\;0 & \cdots \\ \underset{a\;\mathrm{round}}{\underset{︸}{\begin{array}{cccc} 1 & \;0 & \cdots & 0 \end{array}}} & \underset{a\;\mathrm{round}}{\underset{︸}{\begin{array}{cccc} 0 & \cdots & 1 & 0 \end{array}}} & \cdots \end{array}\right).$$

In a round, there is only one 1 in a row while the others in the row are zeros and the sampling process can be formulated as:

(8) B=Q∘X,

where ∘ represents the Hadamard product of two matrices. Namely, $B_{ij}=Q_{ij}\times X_{ij}$. To recover the original data *X* from the observed data matrix *B*, based on its low-rank property, intuitively, we consider the rank minimization problem as

(9) $$\begin{array}{c} \min_{X}\left\{\mathrm{rank}(X),\;\;\mathrm{subject}\;\mathrm{to}\;B=Q\circ X\right\} \end{array}$$

with rank(X) being the rank of *X*. However, this problem is NP-hard [18]. Thus, people turn to solve its convex approximation [10] which reads as

(10) $$\begin{array}{c} \min_{X}\left\{{∥X∥}_{*},\;\;\mathrm{subject}\;\mathrm{to}\;B=Q\circ X\right\}, \end{array}$$

where ${∥X∥}_{*}$ is the nuclear norm which is defined as sum of all singular values of *X*. Rather than directly solving Equation (10), we focus on its penalty problem, by employing positive parameters λ,μ as

(11) $$\begin{array}{c} \min_{X}\left\{\Phi \left(X\right):=\frac{1}{2}{∥Q\circ X-B∥}^{2}+{\lambda ∥X∥}_{*}+\frac{\mu }{2}{∥X∥}_{F}^{2}\right\}. \end{array}$$

The term ${\mu ∥X∥}_{F}^{2}$ is actually the Tikhonov regularization [19] which is developed based on the idea that *Q* is ill-posed. In fact, various nonconvex low-rank regularizations are also developed [20,21,22,23]. However, here, we just consider the convex regularizations and then we can use theory-proved acceleration techniques. The convex composite optimization structure of this problem allows us to apply the forward-backward splitting method [24]. The proximal map [25] of ${\lambda ∥X∥}_{*}$ is SVT operator [10]

(12) $$\begin{array}{c} S_{\tau }\left(X\right)=U\mathrm{Diag}\left\{{\left(\sigma_{i}-\tau \right)}_{+}\right\}V \end{array}$$

where U,V and $\sigma_{i}$ comes from the singular value decomposition of $X=U\mathrm{Diag}\{\left(\sigma_{i}\right)\}V$. Please note that the gradient of $\frac{1}{2}{∥Q\circ X-B∥}^{2}+\frac{\mu }{2}{∥X∥}^{2}$ can be easily calculated as

$$\begin{array}{c} \nabla \left(\frac{1}{2}{∥Q\circ X-B∥}^{2}+\frac{\mu }{2}{∥X∥}^{2}\right)=Q\circ X-Q\circ B+\mu X. \end{array}$$

Obviously, the gradient is Lipschitz with 1+μ. Direct use of forward-backward splitting method to (11) yields the following scheme

(13) $$\begin{array}{c} X^{k+1}=S_{\gamma \lambda }\left(X^{k}-\gamma \left(Q\circ X^{k}-Q\circ B+\mu X^{k}\right)\right), \end{array}$$

where γ is the stepsize. It has been well-known that the forward-backward splitting can be accelerated by the Nesterov technique which is frequently used in sparse or low-rank signal processing [26,27,28,29,30,31,32]. By introducing an auxiliary positive sequence ${\left(t_{k}\right)}_{k\geq 0}$ with $t_{0}=1$ and ${\left(Y^{k}\right)}_{k\geq 0}$, the accelerated scheme can be described as: for any given $X^{0}$, set $Y^{0}=X^{0}$, $t_{0}=1$,

(14a) $$\begin{array}{cc} X^{k+1} & =S_{\gamma \lambda }\left(Y^{k}-\gamma \left(Q\circ Y^{k}-Q\circ B+\mu X^{k}\right)\right), \end{array}$$

(14b) $$\begin{array}{cc} t_{k+1} & =\frac{1+\sqrt{t_{k}^{2}+1}}{2}, \end{array}$$

(14c) $$\begin{array}{cc} Y^{k+1} & =X^{k}+\frac{t_{k}-1}{t_{k+1}}\left(X^{k}-X^{k-1}\right). \end{array}$$

The iterations stop when *k* exceeds the maximum number of iterations, $∥X^{k+1}-X^{k}{∥}_{F}/{∥X^{k}∥}_{F}$ is smaller than a predefined tolerance parameter ϵ, or $∥B-Q\circ X^{k+1}{∥}_{F}/{∥B∥}_{F}$ is smaller than ϵ.

Both Equations (14b) and (14c) just involve basic linear algebra computations. The only costly part for computation is Equation (14a) which needs the SVD computing. Compared with the forward-backward splitting method convergence with speed $O(\frac{1}{k})$, the fast SVT Equation (14) can improve the speed to $O(\frac{1}{k^{2}})$ [27], where *k* denotes the iterations. According to (Theorem 4.4, [27]), if setting $\gamma =\frac{1}{1+\mu }$, we immediately get the following result.

**Proposition** **1.** Assume that ${\left(X^{k}\right)}_{k\geq 0}$ generated by the FSVT Equation (14) minimizing Φ, and $X^{*}$ is any solution to Equation (11). If $\gamma =\frac{1}{1+\mu }$, then it holds that

(15) $$\begin{array}{c} \Phi \left(X^{k}\right)-\Phi \left(X^{*}\right)\leq \frac{2\left(1+\mu \right)∥X^{0}-X^{*}{∥}^{2}}{{\left(k+1\right)}^{2}} \end{array}$$

*with $X^{0}$ being any starting point.*

## 5. Performance Evaluation

The experiments are conducted on MATLAB platform with real-world data sets to evaluate the performance of the proposed method and other data-collection methods. We firstly introduce the experiment environments and parameters setting, and then show the results and analysis of the experiments.

### 5.1. Experiment Environments and Parameters Setting

#### 5.1.1. Real-world Data Sets

Two sets of data from two institutes are used for simulations to evaluate the performance of data-collection methods, one of which is often used in previous WSNs data-collection works [7,9,33], the other one is generated recently [34].

**Figure 3:**
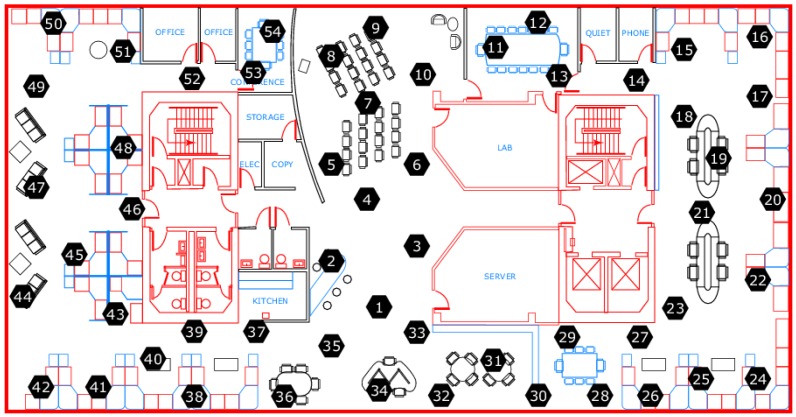
The venue of sensors deployment from Intel Berkeley Research Lab.

The first dataset is a real-world trace from the Intel Berkeley Research Lab and the temperature data collected by 54 sensor nodes on 1 March 2004 [35] are selected. The sensors sense the temperature once every 31 s and the real-world trace forms the $X\in R^{54\times 2880}$ which represent the temperature values sampled by 54 nodes in one day. The sensors are arranged in the Intel Berkeley Research Lab and the venue of sensors deployment is shown in Figure 3.

**Figure 4:**
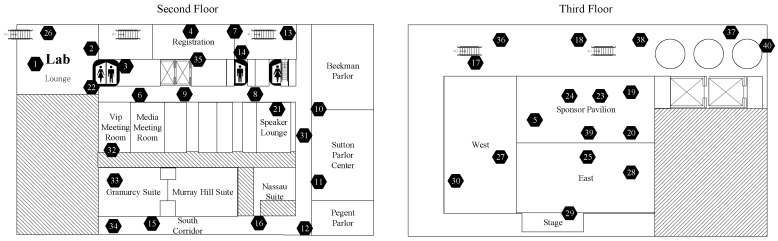
The venue of sensors deployment from Data-Sensing Lab.

The second dataset is the sensing mote data from the Data-Sensing Lab [34]. The dataset is named as the Strata New York 2012 held at the New York Hilton Midtown generated in October 2012 in New York, NY and the temperature humidity data are selected. There are 40 sensors and 1724 time slots forming the matrix $X\in R^{40\times 1724}$. The brief map indicating the position of each sensor mote is shown in Figure 4.

The hexagon with number inside in Figure 3 and Figure 4 represents different sensor nodes with number ID. It is easily seen from the two brief maps that the environment of sensors deployment is usually complex. So, using the basic sparse sampling method will cause some unnecessary energy consumption or demand strict topology as some sensor nodes do not need to transmit data. However, with the proposed RRSS method, all sensor nodes are active at the end of a round and only transmit one value to the sink so that any protocols and routings can be used.

From the sensor nodes of the above two labs, the sensing temperature data are obtained and are formed as two matrices, $X\in R^{54\times 2880}$ form Intel Berkeley Research Lab and $X\in R^{40\times 1724}$ from Data-Sensing Lab. However, with the disturbance when sensing and transmitting, there are many missing data in the two matrices. Actually, the real data curve should be smooth and continuous. To evaluate the performance better and according to the characteristic of WSNs data, the missing measurements are preprocess through the 2D Median filter with the 3×3 box. The sensed measurements are remained the same to preserve the characteristic of the original data to the greatest extent.

#### 5.1.2. Parameters Setup

To evaluate the accuracy of the reconstructed data, the Normalized Mean Absolute Error (NMAE) is used to measure the recovery performance which is defined as:

(16) $$\mathrm{NMAE}=\frac{\sum_{i,j:(i,j)\notin \Omega }|{\hat{X}}_{ij}-X_{ij}|}{\sum_{i,j:(i,j)\notin \Omega }|X_{ij}|},$$

where Ω is the set of sampling values in *X* and the $\hat{X}$ is the recovered data. Using NMAE, only the error of data which are not sampled is considered as the samples are obtained directly. NMAE is used in many previous data-collection works for WSNs [8,9] and is suitable to evaluate the performance of data-collection methods.

The methods are tested over different overall sampling ratios *p*. *p* determines the number of effective value of the measurements matrix. The data recovery experiments are conducted with the sampling ratio changing from 5% to 50%. The traditional random sampling method based on MC is used. With a fixed *p*, through a random selection process of 1-value elements in *Q*, the sampling matrix *Q* can be obtained so that the sampling process is done according to the Equation (2). The proposed RRSS method generates the *Q* with the varying *p* according to the Equation (4).

For the reconstruction methods, the iteration number threshold is set as 1000. The parameters in the Equation (13) are set as λ=0,1,μ=0.1. The stepsize γ is influenced by the matrix *Q* and will be adjust for different data sets. The initial matrix of *X*, $X^{0}$ is a N×T matrix containing pseudorandom values drawn from the standard uniform distribution on the open interval (0 1). The predefined tolerance parameter ϵ for the algorithm to be terminated is set as $\epsilon =e^{-5}$.

### 5.2. Results and Analysis of Experiments

**Figure 5:**
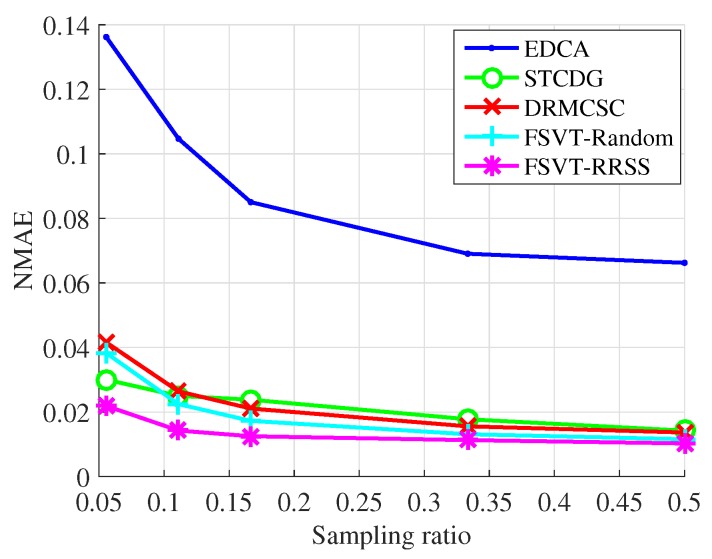
NMAE over sampling ratio for the temperature from Intel Berkeley Research Lab.

**Figure 6:**
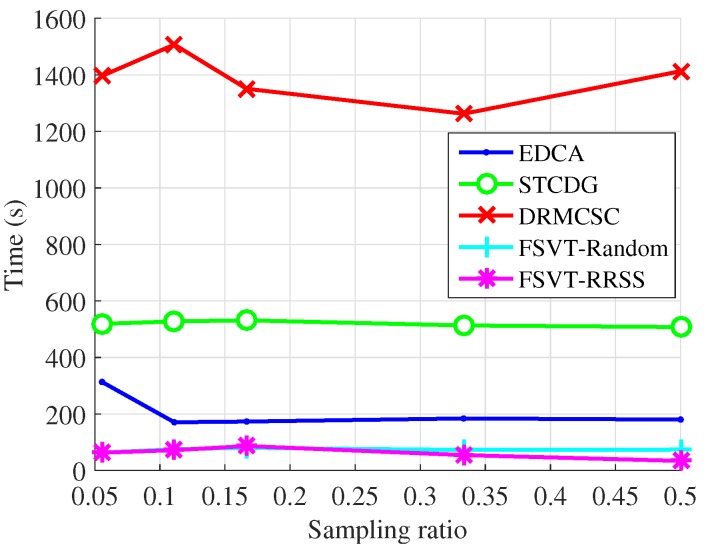
Running time over sampling ratio for the temperature from Intel Berkeley Research Lab.

**Figure 7:**
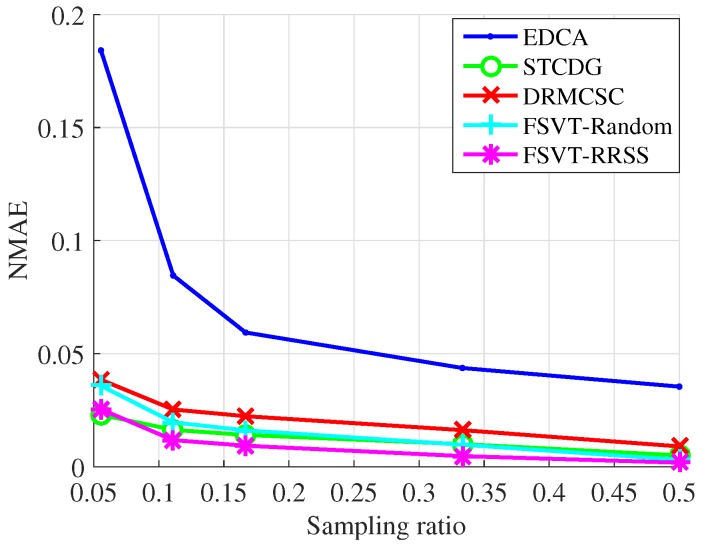
NMAE over sampling ratio for the temperature from Data-Sensing Lab.

**Figure 8:**
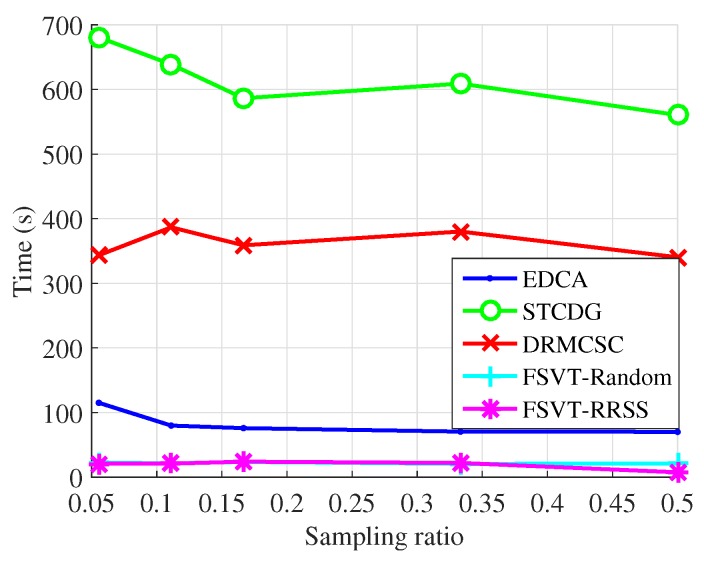
Running time over sampling ratio for the temperature from Data-Sensing Lab.

The experiments on the recovery performance of the proposed data-collection method compared with the EDCA, STCDG and DRMCSC methods are implemented. Moreover, the traditional sampling method with the FSVT algorithm and the proposed RRSS method with the FSVT algorithm are also conducted, which are labeled as FSVT-Random and FSVT-RRSS. The recovery performance includes the recovery accuracy using the metric NMAE and the running time of the algorithm. In each experiment, the process of randomly sampling data and recovering data was repeated 10 times. The experimental results presented were the mean NMAE and mean running time, which are shown in Figure 5, Figure 6, Figure 7 and Figure 8.

Figure 5 and Figure 6 show the experimental results of the recovery performance for the temperature data from Intel Berkeley Research Lab. It can be seen that the proposed method, the FSVT reconstruction algorithm with the RRSS method, outperforms other methods in both reconstruction accuracy and time especially when the sampling ratio is low. Not only the RRSS sampling method enhances the FSVT method, improving the reconstruction accuracy, but also it decreases the running time as shown in Figure 6.

Figure 7 and Figure 8 show the experimental results of the recovery performance for the temperature data from Data-Sensing Lab. The results are similar to that of the data from Intel Berkeley Research Lab, but the recovery accuracy is not as good as that, overall. Actually, the stepsize γ of the FSVT algorithm in the Equation (13) decreases as it is more difficult to find the correct solution compared with that for the data from Intel Berkeley Research Lab. The FSVT algorithm is more suitable for large-scale WSNs although the proposed RRSS method can improve its ability in solving the recovery problem with small size data.

It is seen in Figure 6 and Figure 8 that there is little change in running time of a method with increasing sampling ratios. We think this is because the iteration number of each algorithm reaches the maximum, which is set as 1000 for all methods. The stopping criterion has been described above. Because the time length of each iteration is close, the total running time of an algorithm to recover the original data is close with different sampling ratios.

**Figure 9:**
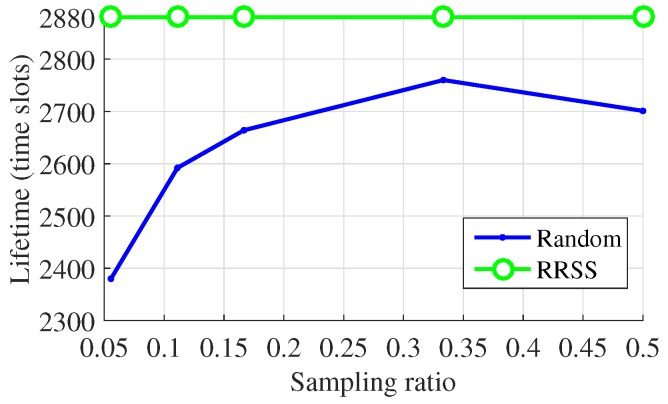
Lifetime over sampling ratio for WSNs from Intel Berkeley Research Lab.

**Figure 10:**
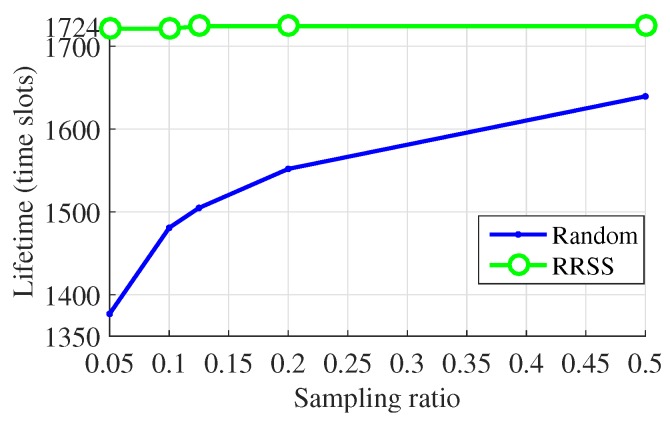
Lifetime over sampling ratio for WSNs from Data-Sensing Lab.

Moreover, the performance of the RRSS method for the reduction of energy consumption is also evaluated. Here we use the network lifetime as the metric. The power consumption model adopted in our study and the definition of the network lifetime are similar to those in previous works [7,8]. The lifetime of a network is in accordance with the time slot of the first node running out of its energy. The energy consumption is simplified as the packages of transmitting data. To show the performance of methods more intuitively, we regard the number of data package that each sensor node senses in a time slot and transmits in the transmission period as one. In addition, we set the death threshold as N×p. It is the purpose of the sampling methods that the network can live during the whole time slots and the WSN will not break ahead of time. However, when using the traditional random sampling method, some nodes die ahead of time. The proposed sampling method makes every sensor sense and transmit data at the same times in each round, i.e., the network reaches load balance. Because of this advantage, the network can live as expected with the RRSS method as shown in Figure 9 and Figure 10.

**Figure 11:**
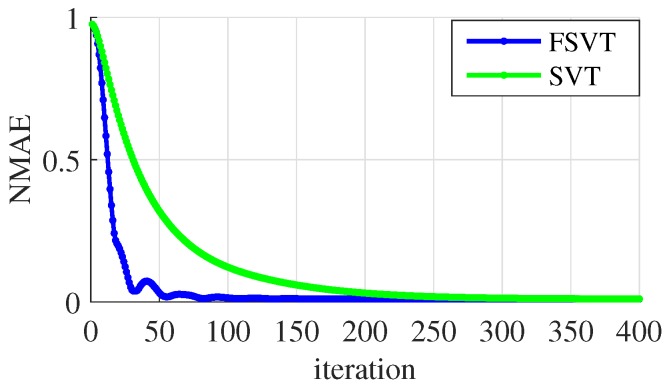
NMAE over iteration time between FSVT and SVT.

Figure 11 shows the accelerated speed of the FSVT method compared with the original SVT method. The reconstruction error of FSVT method is close to the minimum when the iteration number is 100 while that is more than 200 for the original SVT method, i.e., the FSVT algorithm can be twice faster than the original SVT algorithm, so the FSVT algorithm is used here.

## 6. Conclusions

In this paper, we propose a novel data-collection method based on MC for large-scale WSNs. We design a RRSS method, in which sensor nodes are selected randomly and uniformly and are in the sleep mode most of the time to greatly reduce the energy consumption. From the samples obtained by the proposed sampling method, a corresponding FSVT algorithm is given to recover the data accurately and fast. The experiments are conducted with two real-world data sets and the simulation results show that the proposed sampling method can enhance the corresponding algorithm. Moreover, the proposed data-collection method outperforms other methods in terms of the reconstruction accuracy and rate. Our future work can be conducted form two aspects: combining our sampling method with other data gathering methods to further reduce the energy consumption and dealing with robust MC problem of WSNs with our method.
